# Dual Metabolic Targeting of Cancer: Lessons from Metformin and 2-Deoxy-D-Glucose Combinations

**DOI:** 10.3390/cancers18162537

**Published:** 2026-08-07

**Authors:** Vesna Zeljković, Slaviša Minić, Marko Mladenović, Dejan Milenković, Zoran Marković, Tanja V. Soldatović, Vanja Kunkin, Maja Karaman

**Affiliations:** 1 Department of Biomedical Sciences, State University of Novi Pazar, Vuka Karadžića 9, 36300 Novi Pazar, Serbia; sminic@np.ac.rs; 2 Department of Sciences and Mathematics, State University of Novi Pazar, Vuka Karadžića 9, 36300 Novi Pazar, Serbia; markohem87@gmail.com (M.M.); zmarkovic@uni.kg.ac.rs (Z.M.); tsoldatovic@np.ac.rs (T.V.S.); 3 Department of Chemistry, Faculty of Sciences and Mathematics, University of Niš, Višegradska 33, 18000 Niš, Serbia; 4 Department of Science, Institute of Information Technologies, University of Kragujevac, Liceja Kneževine Srbije 1A, 34000 Kragujevac, Serbia; dejanm@uni.kg.ac.rs; 5 Đorđe Joanović Zrenjanin General Hospital, Dr Vase Savića br. 5, 23000 Zrenjanin, Serbia; kunkinbre@gmail.com; 6 Department of Biology and Ecology, Faculty of Sciences, University of Novi Sad, Trg Dositeja Obradovića 2, 21000 Novi Sad, Serbia; maja.karaman@dbe.uns.ac.rs

**Keywords:** 2-deoxy-D-glucose, cancer metabolism, glycolysis, metabolic targeting, metabolic plasticity, metformin, molecular docking

## Abstract

**Background**: Metabolic reprogramming enables cancer cells to adapt to energetic stress and sustain proliferation. Simultaneous inhibition of glycolysis and mitochondrial respiration has emerged as a promising strategy to overcome metabolic plasticity. This study evaluated the antiproliferative effects of the glycolytic inhibitor 2-deoxy-D-glucose (2-DG) alone and in combination with metformin in human cancer cell lines. **Methods**: Human cervical carcinoma (*HeLa*), lung adenocarcinoma (*A549*), colorectal adenocarcinoma (*HT-29*), and normal lung fibroblasts (*MRC-5*) were treated with 2-DG or metformin individually, or with metformin in the presence of a fixed concentration of 2-DG (1 mM). Cell viability was assessed using the sulforhodamine B assay after 24 and 48 h. IC_50_ values were calculated by nonlinear regression, Dose Reduction Index (DRI) analysis evaluated sensitization to metformin, and molecular docking was performed to investigate interactions with selected metabolic targets. **Results**: Both 2-DG and metformin inhibited cell proliferation in a concentration- and time-dependent manner. *HeLa* cells were the most sensitive to glycolytic inhibition, while *A549* and *HT-29* cells showed moderate susceptibility. Co-treatment with 2-DG significantly enhanced metformin activity, reducing its IC_50_ in *HeLa* cells from 6.04 to 2.00 mM after 24 h and from 2.28 to 1.56 mM after 48 h. DRI analysis demonstrated increased sensitivity to metformin in all cancer cell lines, particularly *HT-29* and *A549*, whereas normal *MRC-5* fibroblasts remained comparatively less affected. Molecular docking revealed favorable binding of both 2-DG and metformin to proteins involved in cellular energy metabolism. **Conclusions**: Combined inhibition of glycolysis and mitochondrial respiration potentiates the antiproliferative effects of metformin, increases metabolic vulnerability in cancer cells, and is supported by molecular docking evidence of interactions with metabolic targets, while showing limited toxicity toward normal fibroblasts. These findings support dual metabolic targeting as a promising therapeutic strategy and warrant further mechanistic and in vivo studies.

## Simple Summary

Cancer cells require large amounts of energy to sustain their rapid growth and survival. Unlike normal cells, they often rely on altered metabolic pathways, making energy metabolism an attractive target for the development of new anticancer therapies. In this study, we investigated whether combining metformin, a widely used antidiabetic drug, with 2-deoxy-D-glucose, a compound that blocks glucose utilization, could more effectively inhibit the growth of cancer cells than either treatment alone. Using cervical, lung, and colorectal cancer cell lines, we found that the combined treatment reduced cancer cell growth more efficiently than single agents while having a comparatively weaker effect on normal human cells. These findings suggest that simultaneously targeting two major energy-producing pathways may improve the anticancer activity of metformin and support further preclinical studies aimed at developing metabolism-based therapeutic strategies for cancer treatment.

## 1. Introduction

Cancer remains one of the leading causes of morbidity and mortality worldwide despite substantial advances in diagnosis and treatment. One of the hallmarks of cancer is metabolic reprogramming, which enables tumor cells to sustain rapid proliferation, survive under adverse microenvironmental conditions, and resist conventional therapies [1,2]. Unlike normal cells, many cancer cells exhibit increased glucose uptake and preferential conversion of glucose to lactate even in the presence of adequate oxygen levels, a phenomenon known as the Warburg effect [3]. This metabolic adaptation provides both energy and biosynthetic intermediates required for continuous cell growth and division, making glucose metabolism an attractive therapeutic target in oncology [4].

The enhanced glycolytic activity of tumor cells is accompanied by overexpression of glucose transporters (GLUTs), increased activity of glycolytic enzymes, and activation of signaling pathways involved in cellular energy sensing and nutrient utilization [5,6]. Furthermore, hypoxic regions within tumors stimulate the expression of hypoxia-inducible factor-1 alpha (HIF-1α), which promotes glycolysis and suppresses mitochondrial oxidative metabolism, thereby facilitating tumor progression and adaptation to metabolic stress [7]. Given the central role of glucose metabolism in cancer cell survival, therapeutic strategies targeting tumor bioenergetics have gained considerable attention in recent years [8].

Metformin, a widely prescribed antidiabetic drug, has emerged as a promising anticancer agent due to its ability to interfere with cellular energy metabolism [9,10]. Numerous experimental studies have demonstrated that metformin inhibits cancer cell proliferation, induces cell-cycle arrest, and promotes apoptosis in a variety of tumor types [11,12,13]. The primary mechanism underlying these effects involves activation of AMP-activated protein kinase (AMPK), followed by inhibition of the mammalian target of rapamycin (mTOR) signaling pathway [14,15].

Several in vitro studies have demonstrated that metformin exerts significant antitumor activity in leukemia, prostate, breast, endometrial, colorectal, and lung cancer models [11,12,13,16]. In myeloid leukemia cells, metformin induces G1 cell-cycle arrest through downregulation of cyclin D1 and Mcl-1 and upregulation of p21, Bax, caspase-3, caspase-9, and PARP-1 expression [11]. In prostate cancer cells, metformin has been shown to promote apoptosis through AMPK–p53 signaling and suppression of cell-cycle progression [17]. Studies performed on endometrial cancer cells suggest that metformin-induced cell death may also occur through caspase-independent mechanisms, indicating the involvement of alternative pathways of programmed cell death [18].

Another promising metabolic inhibitor is 2-deoxy-D-glucose (2-DG), a synthetic glucose analogue that differs from glucose by the absence of a hydroxyl group at the 2′ position of the pyranose ring [19]. Following cellular uptake via GLUT transporters, 2-DG is phosphorylated by hexokinase to form 2-DG-6-phosphate but cannot undergo further glycolytic metabolism [20]. Consequently, 2-DG accumulates intracellularly and inhibits glycolysis, leading to depletion of ATP, disruption of cellular bioenergetics, and activation of metabolic stress responses [20,21].

Numerous studies have demonstrated that 2-DG suppresses proliferation, migration, and invasion while inducing apoptosis and cell-cycle arrest in various cancer cell lines [17,22,23]. These effects have been associated with downregulation of anti-apoptotic proteins such as Mcl-1 and Bcl-2, inhibition of cathepsin L activity, and modulation of the AMPK/mTOR signaling pathway [17,22,23,24].

Because metformin primarily targets mitochondrial oxidative phosphorylation whereas 2-DG inhibits glycolysis, their combined application may simultaneously disrupt the two major pathways responsible for cellular energy production [25]. Such dual metabolic targeting has been proposed as an effective strategy for overcoming metabolic plasticity, a characteristic feature of cancer cells that enables adaptation to metabolic stress and therapeutic interventions [26]. Previous studies have reported enhanced antiproliferative and pro-apoptotic effects of metformin and 2-DG in several tumor models, including prostate cancer, suggesting that combined metabolic inhibition may produce greater antitumor activity than either agent alone [17,25,27].

Despite growing evidence supporting the anticancer potential of metformin and 2-deoxy-D-glucose, the effects of their combined application remain insufficiently characterized in several human tumor cell lines, particularly in cervical, lung, and colorectal cancer models. Our previous investigations demonstrated that combinations of metformin with caffeine, as well as nitroglycerin-based metabolic combinations involving caffeine and 2-deoxy-D-glucose, exerted antiproliferative effects in several cancer cell models [28,29]. These findings further support the concept that simultaneous targeting of multiple metabolic pathways may represent a promising strategy for enhancing anticancer efficacy. To further explore this concept, cervical, lung, and colorectal cancer cell lines were selected as experimental models because they represent biologically distinct malignancies characterized by substantial metabolic heterogeneity. Furthermore, simultaneous inhibition of glycolysis and mitochondrial respiration may enhance energetic stress and activate signaling pathways associated with apoptosis and growth suppression, including AMPK-dependent mechanisms. Therefore, the aim of the present study was to evaluate the effects of metformin and 2-deoxy-D-glucose, administered individually and in combination, on the viability and proliferative capacity of *HeLa* cervical carcinoma, *A549* lung adenocarcinoma, and *HT29* colorectal adenocarcinoma cells, while simultaneously assessing their effects on the normal human lung fibroblast cell line *MRC-5*.

## 2. Materials and Methods

### 2.1. Cell Lines and Culture Conditions

*HeLa* (human cervical carcinoma; ATCC^®^ CCL-2™), *A549* (human lung adenocarcinoma; ATCC^®^ CCL-185™), *HT-29* (human colorectal adenocarcinoma; ATCC^®^ HTB-38™), and *MRC-5* (normal human lung fibroblasts; ATCC^®^ CCL-171™) cell lines were purchased from the American Type Culture Collection (ATCC, Manassas, VA, USA). To ensure unambiguous identification and experimental reproducibility, the respective accession numbers from the Cellosaurus knowledge resource are provided as follows: *HeLa* (CVCL_0030), *A549* (CVCL_0023), *HT-29* (CVCL_0320), and *MRC-5* (CVCL_0440). Cells were maintained in Dulbecco’s Modified Eagle Medium (DMEM; Sigma-Aldrich, St. Louis, MO, USA) supplemented with 10% fetal bovine serum (FBS), 100 U/mL penicillin, and 100 μg/mL streptomycin at 37 °C in a humidified atmosphere containing 5% CO_2_. Cells were routinely passaged at approximately 80–90% confluence and used during the exponential growth phase. For viability assays, cells were seeded into 96-well plates at a density of 1 × 10^4^ cells/well and allowed to attach for 24 h before treatment.

### 2.2. Reagents

Metformin hydrochloride (99.99%; Galenika, Belgrade, Serbia) was dissolved in phosphate -buffered saline (PBS; Dulbecco’s PBS, Capricorn Scientific GmbH, Ebsdorfergrund, Germany) immediately before use. 2-Deoxy-D-glucose (2-DG; Abcam, Cambridge, UK) was dissolved in PBS immediately before use. Sulforhodamine B (SRB), Tris base, dimethyl sulfoxide (DMSO), and Dulbecco’s Modified Eagle Medium (DMEM) were obtained from Sigma-Aldrich (St. Louis, MO, USA). Trichloroacetic acid (TCA) was purchased from Merck (Darmstadt, Germany), whereas acetic acid was obtained from Zorka Pharma Hemija (Šabac, Serbia).

Stock solutions were prepared according to the manufacturers’ instructions and diluted in complete culture medium immediately before use. The final concentration of DMSO did not exceed 0.1% (*v*/*v*) in any treatment condition.

### 2.3. Cell Viability and Proliferation Assays

#### 2.3.1. Trypan Blue Exclusion Assay

Before initiating the treatments, cell viability was verified using the Trypan Blue exclusion assay [30]. Cell samples were exposed to 0.1% Trypan Blue, and viable cells (excluding the dye) as well as non-viable cells (taking up the dye) were quantified using a Neubauer hemocytometer and an inverted microscope. Cell cultures with viability exceeding 95% were considered suitable for further experimental analyses.

#### 2.3.2. Sulforhodamine B (SRB) Assay

The antiproliferative effects of metformin and 2-deoxy-D-glucose (2-DG), administered individually or in combination, were evaluated using the sulforhodamine B (SRB) assay. Cells were seeded into 96-well plates at a density of 1 × 10^4^ cells/well and allowed to attach for 24 h. Subsequently, cells were treated with a range of concentrations of metformin (0.05–50 mM) and 2-deoxy-D-glucose (2-DG; 0.05–20 mM) alone or in combination, for 24 and 48 h.

At the completion of the treatment period, cells were fixed with ice-cold 10% (*w*/*v*) trichloroacetic acid for 60 min at 4 °C. After fixation, the plates were rinsed with distilled water and incubated with 0.4% (*w*/*v*) sulforhodamine B solution prepared in 1% acetic acid for 30 min at room temperature. Excess dye was eliminated by repeated washing with 1% acetic acid, while the retained dye was released using 10 mM Tris base solution (pH 10.5). The absorbance values were determined at 540 nm using a Synergy HTX microplate reader (BioTek Synergy HTX, Agilent Technologies, Santa Clara, CA, USA).

Cell viability was expressed as a percentage of the untreated control, which was defined as 100% viability. IC_50_ values were calculated from dose–response curves generated by nonlinear regression analysis using GraphPad Prism 8.0 [31,32]. Combination treatments were performed using metformin dose–response curves in the presence of a fixed 2-DG concentration (corresponding to 50% of its previously determined IC_50_ value, for 24 or 48 h).

### 2.4. Molecular Docking Analysis

Molecular docking was performed using AutoDock-GPU [33] with AutoGrid-generated grid maps. The three-dimensional structures of the selected protein targets were retrieved from the RCSB Protein Data Bank (PDB). The targets were selected to represent key mechanistic nodes relevant to the experimental design: human AMP-activated protein kinase (AMPK; PDB ID: 4CFE), human mechanistic target of rapamycin kinase (mTOR; PDB ID: 4JSV), and human caspase-3 (PDB ID: 2H5I). These structures were used to represent energy-sensing and metformin-associated signaling, downstream mTOR-dependent growth regulation, and apoptosis-related response, respectively [34,35,36].

Protein structures were prepared by removing crystallographic water molecules and non-relevant heteroatoms. When available, the position of the co-crystallized/native ligand was used to define the primary docking grid for the initial 2-DG docking. For the caspase-3 structure 2H5I, only chain A was retained for docking in order to avoid artificial inclusion of additional crystallographic chains. Receptor structures were prepared in PDBQT format using AutoDock Tools/MGL Tools v1.5.7, and AutoGrid v4.2.6 maps were generated with a grid spacing of 0.375 Å [37]. The exhaustiveness parameter was set to 8, and the docking protocol was performed with 100 independent runs.

The three-dimensional structures of the ligands, metformin and 2-deoxy-D-glucose (2-DG), were obtained through geometry optimization using the Gaussian 16 program at the B3LYP/6-311G** level of theory [38]. The optimized structures were converted to docking input formats and prepared as PDBQT files. To reflect the experimental design in which 2-DG was applied as a sensitizing agent for metformin treatment, a sequential docking protocol was used. First, 2-DG was docked into the selected receptor binding sites. The lowest-energy 2-DG pose was retained as a fixed ligand conformation within the binding site, and metformin was subsequently docked into the resulting protein–2-DG model. Docking parameters were validated by redocking the native ligand into the corresponding binding site, and the obtained RMSD value was used as a measure of protocol reliability. Docking simulations were performed using grid boxes centered on the active sites with dimensions defined according to the corresponding binding pockets. The protonation state of metformin was considered during ligand preparation.

The resulting protein–2-DG–metformin complexes were analyzed based on estimated binding affinity (kcal/mol), ligand binding pose, centroid–centroid distance between the fixed 2-DG pose and the best metformin pose, and interactions with amino acid residues. Docking results were interpreted as supportive in silico evidence for molecular compatibility with the experimentally observed sensitization effect, rather than as direct proof of pharmacological synergy.

### 2.5. Molecular Dynamics Simulations

To assess the post-docking stability of the ternary protein-ligand complexes, molecular dynamics (MD) simulations were performed for the 4CFE, 4JSV, and 2H5I protein-2-DG-metformin systems using GROMACS 2026.1 [38]. The docking-derived protein-2-DG-metformin complexes were used as starting structures for the simulations. Trajectories were processed by removing periodic boundary condition artifacts and solvent molecules, followed by least-squares fitting to the protein backbone.

The structural stability and flexibility of the simulated complexes were evaluated using standard trajectory descriptors: root-mean-square deviation (RMSD), radius of gyration (Rg), and root-mean-square fluctuation (RMSF). RMSD was calculated for the protein backbone after backbone fitting, Rg was used to evaluate global compactness, and RMSF was calculated per residue to identify flexible protein regions. The total simulation time for each complex was 100 ns. Systems were parameterized using an appropriate biomolecular force field (CHARMM36m), solvated in an explicit water environment, neutralized with counterions, energy minimized, and equilibrated under NVT and NPT conditions before production runs.

### 2.6. Statistical Analysis

Statistical analyses were based on data obtained from three independent biological experiments, with each condition evaluated in three replicate wells. The results are presented as mean values with standard deviations (SD). All statistical calculations were performed using GraphPad Prism software (version 8.0; GraphPad Software, San Diego, CA, USA). Group differences were assessed using one-way ANOVA with Tukey’s multiple comparison procedure. A two-sided *p* value < 0.05 was considered indicative of statistical significance.

The half-maximal inhibitory concentration (IC_50_) values were calculated from dose–effect relationships using nonlinear regression modeling. The interaction profile of metformin and 2-DG combinations was analyzed by applying the Chou–Talalay approach through CalcuSyn software (Version 2.0, Biosoft, Cambridge, UK), according to the experimental design used for the combined treatments. Combination index (CI) values were subsequently used to define the nature of the drug interaction, with CI < 1, CI = 1, and CI > 1 corresponding to synergistic, additive, and antagonistic effects, respectively [39,40].

## 3. Results

To investigate the potential of metabolic targeting in cancer cells, the antiproliferative effects of metformin and 2-deoxy-D-glucose (2-DG), applied either as single agents or in combination, were assessed in *HeLa*, *A549*, and *HT29* cancer cell lines and compared with their effects on normal human lung fibroblasts (*MRC-5*). Cell viability and growth inhibition were determined using the sulforhodamine B (SRB) assay after 24 h and 48 h of exposure.

### 3.1. Effects of 2-Deoxy-D-Glucose

**Figure 1:**
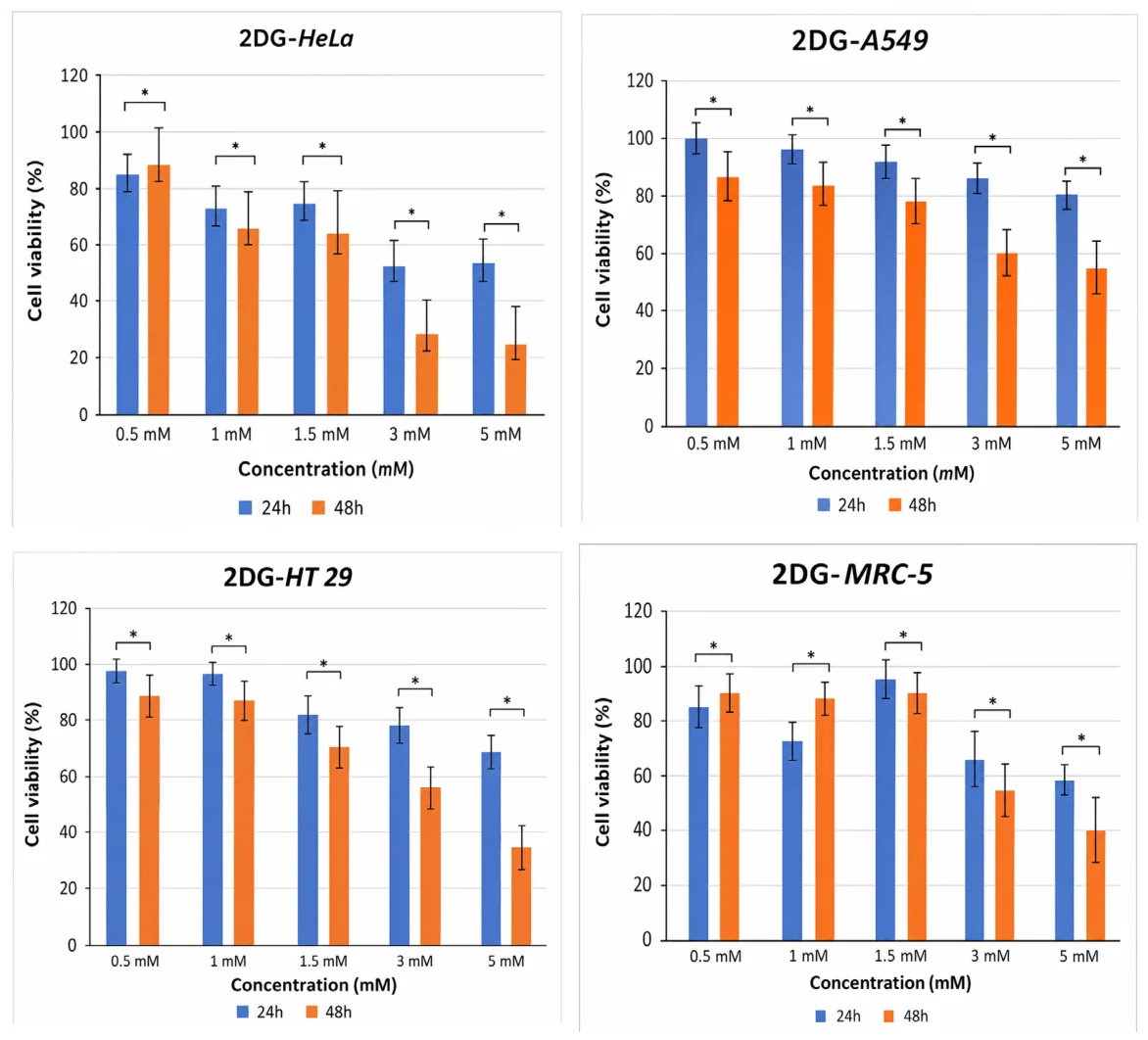
Effect of 2-Deoxy-D-Glucose (2-DG) on the Viability of Human Cancer and Normal Cell Lines. *HeLa*, *A549*, *HT-29*, and *MRC-5* cells were exposed to increasing concentrations of 2-DG (0.5–5 mM) for 24 h and 48 h. Cell viability was determined by the SRB assay and expressed as a percentage of the untreated control. Data are presented as mean ± SD (*n* = 3). * *p* < 0.05.

Treatment with 2-deoxy-D-glucose (2-DG) resulted in a concentration- and time-dependent reduction in cell viability across all examined cancer cell lines. The strongest cytotoxic response was observed in HeLa cells, which exhibited the highest sensitivity to 2-DG treatment. Following 24 h of exposure to 5 mM 2-DG, cell viability decreased to 53.97% of the untreated control. Prolonged exposure markedly enhanced the antiproliferative effect, as reflected by a reduction in the IC_50_ value from 5.45 mM at 24 h to 2.01 mM at 48 h (Figure 1).

*A549* lung adenocarcinoma and *HT-29* colorectal adenocarcinoma cells displayed a more moderate response to 2-DG treatment. Nevertheless, a clear time-dependent increase in cytotoxicity was observed, with greater reductions in cell viability after 48 h compared with 24 h of exposure (Table 1). These findings indicate that the anticancer activity of 2-DG is both concentration- and exposure time-dependent, with *HeLa* cells demonstrating the greatest susceptibility among the tested tumor cell lines.

### 3.2. Combined Treatment with Metformin

**Figure 2:**
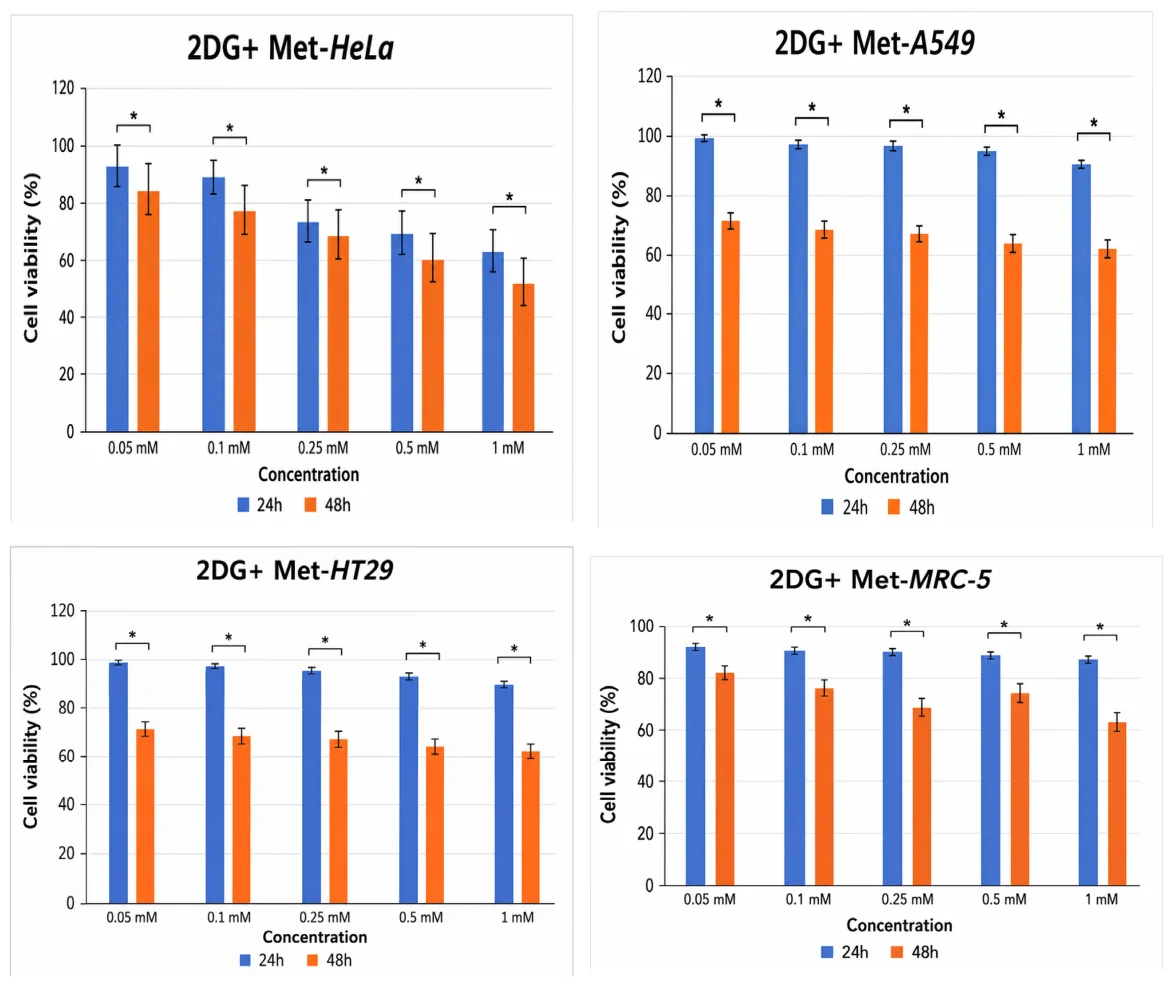
Combined Treatment with 2-Deoxy-D-Glucose and Metformin Reduces Cell Viability in Human Cancer and Normal Cell Lines. *HeLa*, *A549*, *HT-29*, and *MRC-5* cells were exposed to 2-deoxy-D-glucose (2-DG) and increasing concentrations of metformin (0.05–1 mM) for 24 h and 48 h. Cell viability was assessed using the SRB assay and expressed as a percentage of the untreated control. Data are presented as mean ± SD (*n* = 3). * *p* < 0.05.

To investigate whether simultaneous inhibition of glycolysis and mitochondrial respiration could enhance antiproliferative activity, cells were treated with increasing concentrations of metformin in the presence of a fixed concentration of 2-deoxy-D-glucose (2-DG, 1 mM). Combined treatment resulted in a concentration- and time-dependent reduction in cell viability in all examined cell lines (Figure 2).

The strongest response was observed in *HeLa* cells, where the addition of 2-DG markedly enhanced the antiproliferative activity of metformin. Cell viability progressively decreased with increasing metformin concentrations, reaching approximately 60% and 50% of the untreated control after 24 h and 48 h of treatment, respectively. Consistent with these findings, the IC_50_ value of metformin decreased from 6.04 mM to 2.00 mM after 24 h and from 2.28 mM to 1.56 mM after 48 h in the presence of 2-DG.

*A549* and *HT-29* cells also exhibited enhanced sensitivity to the combined treatment, although the magnitude of growth inhibition was less pronounced than that observed in *HeLa* cells. In contrast, *MRC-5* fibroblasts displayed a comparatively weaker response, suggesting a degree of selectivity toward malignant cells. Overall, these results indicate that simultaneous targeting of glycolysis and mitochondrial energy metabolism potentiates the antiproliferative effects of metformin and enhances cancer cell sensitivity to combined metabolic inhibition.

Previously determined IC_50_ values for metformin alone were used for comparison with the combined treatment (Table S1) [28]. *HeLa* cells exhibited the highest sensitivity to metformin, whereas *HT-29* and *MRC-5* cells were less responsive.

Comparison of IC_50_ values obtained for metformin alone and in combination with 2-DG revealed a marked enhancement of antiproliferative activity following dual metabolic targeting. Therefore, fold reduction values were calculated to quantify the increase in metformin efficacy induced by 2-DG (Table 1). Although the greatest overall reduction in cell viability was observed in *HeLa* cells, comparison of IC_50_ values demonstrated the largest fold reduction in *A549* cells. The addition of 2-DG reduced the IC_50_ value of metformin by 7.55-fold after 24 h and 5.44-fold after 48 h of treatment, indicating increased cellular sensitivity to metformin following glycolytic inhibition.

### 3.3. Molecular Docking and Molecular Dynamics

Because metabolic reprogramming and apoptosis regulation represent central determinants of cancer cell response to metabolic stress, AMPK, mTOR kinase, and caspase-3 were selected as representative targets for computational evaluation.

#### 3.3.1. Molecular Docking

Molecular docking was used as an exploratory approach to evaluate the potential compatibility of metformin and 2-DG within structurally relevant metabolic and apoptosis-associated protein environments. Three representative human protein structures were selected: AMPK, mTOR kinase, and caspase-3. For each target, 2-DG was first docked into the selected binding region, and the lowest-energy 2-DG pose was retained as a fixed component of the receptor. Metformin was then docked into the corresponding 2-DG-bound receptor model.

The docking results are summarized in Table 2. 2-DG showed moderate predicted binding energies toward all investigated targets, with binding energies ranging from −3.45 to −3.98 kcal/mol. The most favorable 2-DG docking score was observed for the mTOR kinase structure 4JSV (−3.98 kcal/mol), followed by AMPK 4CFE (−3.65 kcal/mol) and caspase-3 2H5I (−3.45 kcal/mol) (Table 2).

Following the initial docking of 2-DG, metformin was docked into the corresponding preformed protein–2-DG models. In all three targets, the addition of metformin resulted in a more favorable docking energy for the final protein–2-DG–metformin arrangement compared with the initial protein–2-DG docking step. The most favorable energy after metformin docking was obtained for the mTOR kinase model 4JSV (−6.13 kcal/mol), followed by AMPK 4CFE (−5.02 kcal/mol) and caspase-3 2H5I (−4.44 kcal/mol). These results suggest that metformin can be spatially accommodated within 2-DG-bound protein models and provide a structural hypothesis supporting the experimentally observed enhancement of metformin activity. The centroid-centroid distance between metformin and the fixed 2-DG pose was used to evaluate the spatial relationship between the two ligands within the ternary complexes. Importantly, this computational trend is consistent with the experimental observation that 2-DG enhanced the antiproliferative effect of metformin, as reflected by reduced metformin IC_50_ values and favorable DRI profiles in cancer cell lines.

The shortest distance was observed for the AMPK complex 4CFE (7.95 Å), suggesting the most favorable spatial compatibility between the two ligands. In contrast, metformin and 2-DG were positioned farther apart in the mTOR kinase and caspase-3 models, with centroid distances of 14.38 Å and 13.68 Å, respectively. These findings indicate that metformin can be accommodated in 2-DG-bound forms of all investigated protein targets, although the degree of spatial proximity differs among the complexes.

**Figure 3:**
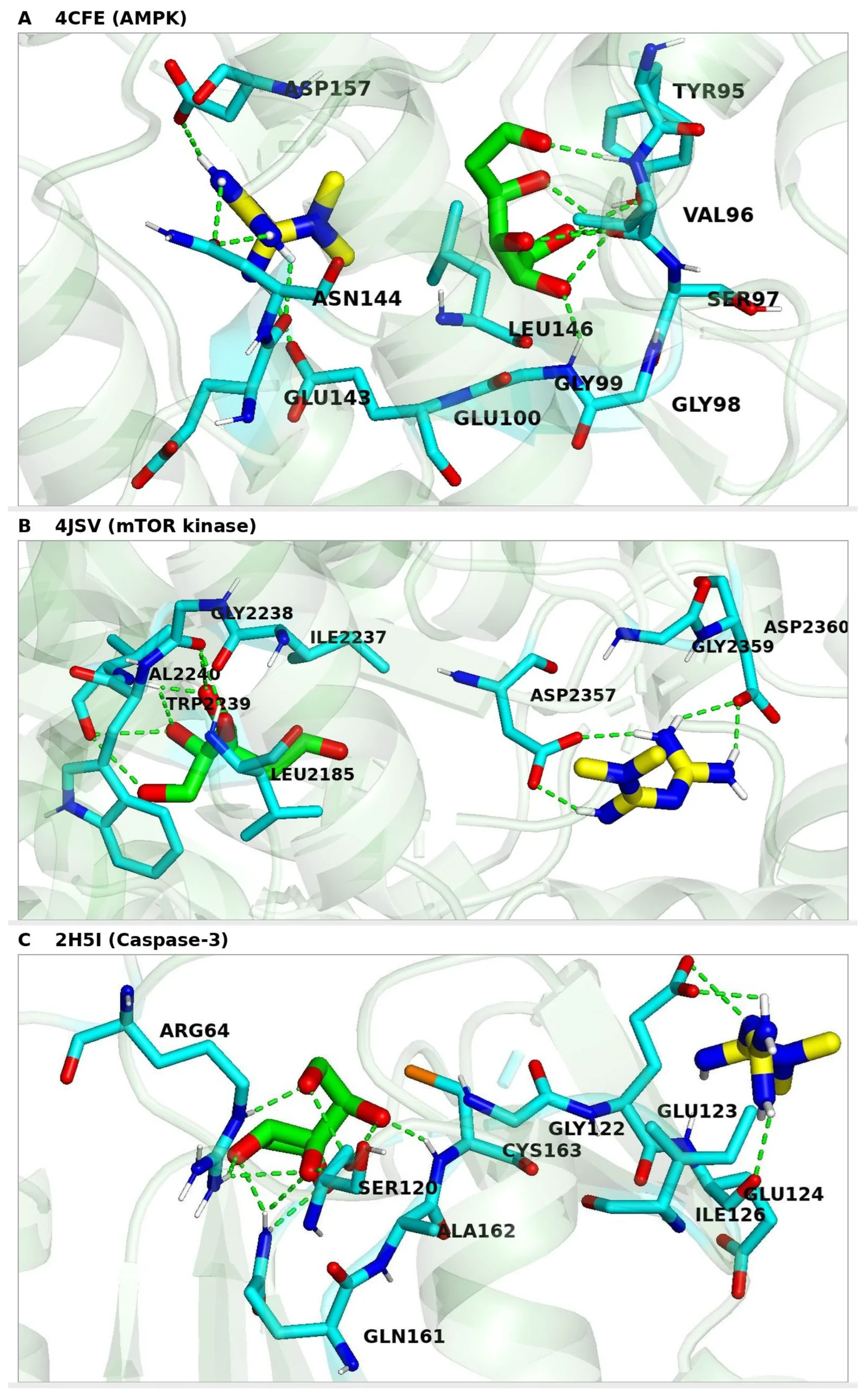
Binding-site interactions of 2DG and metformin (Met) in the selected protein targets after sequential docking. (**A**) 4CFE (AMPK), (**B**) 4JSV (mTOR kinase), and (**C**) 2H5I (caspase-3). 2-DG is shown in green, metformin in yellow, surrounding amino-acid residues in cyan, and polar contacts as green dashed lines.

Visual inspection of the docking-derived binding-site arrangements revealed distinct residue-level interaction patterns for 2DG and metformin (UNK) in the selected protein targets (Figure 3). In the AMPK structure 4CFE, 2DG was positioned in a polar region and formed multiple contacts with residues including TYR95, SER97, GLY99, GLU100, and LEU146, whereas metformin was accommodated in a neighboring region involving GLU143, ASN144, and ASP157. In the mTOR kinase structure 4JSV, 2DG occupied a pocket defined by residues such as LEU2185, ILE2237, GLY2238, TRP2239, and ALA2240, while metformin was located in a spatially distinct polar region involving ASP2357, GLY2359, and ASP2360. In the caspase-3 structure 2H5I, 2DG formed an extensive interaction network with ARG64, SER120, GLN161, ALA162, CYS163, and GLY122, whereas metformin interacted with residues located in the GLU123/GLU124/ILE126 region. These residue-level interaction patterns support the ability of both compounds to be accommodated within biologically relevant protein environments, while also indicating that the two ligands do not necessarily occupy the same local sub-pocket in each target.

#### 3.3.2. Molecular Dynamics

**Figure 4:**
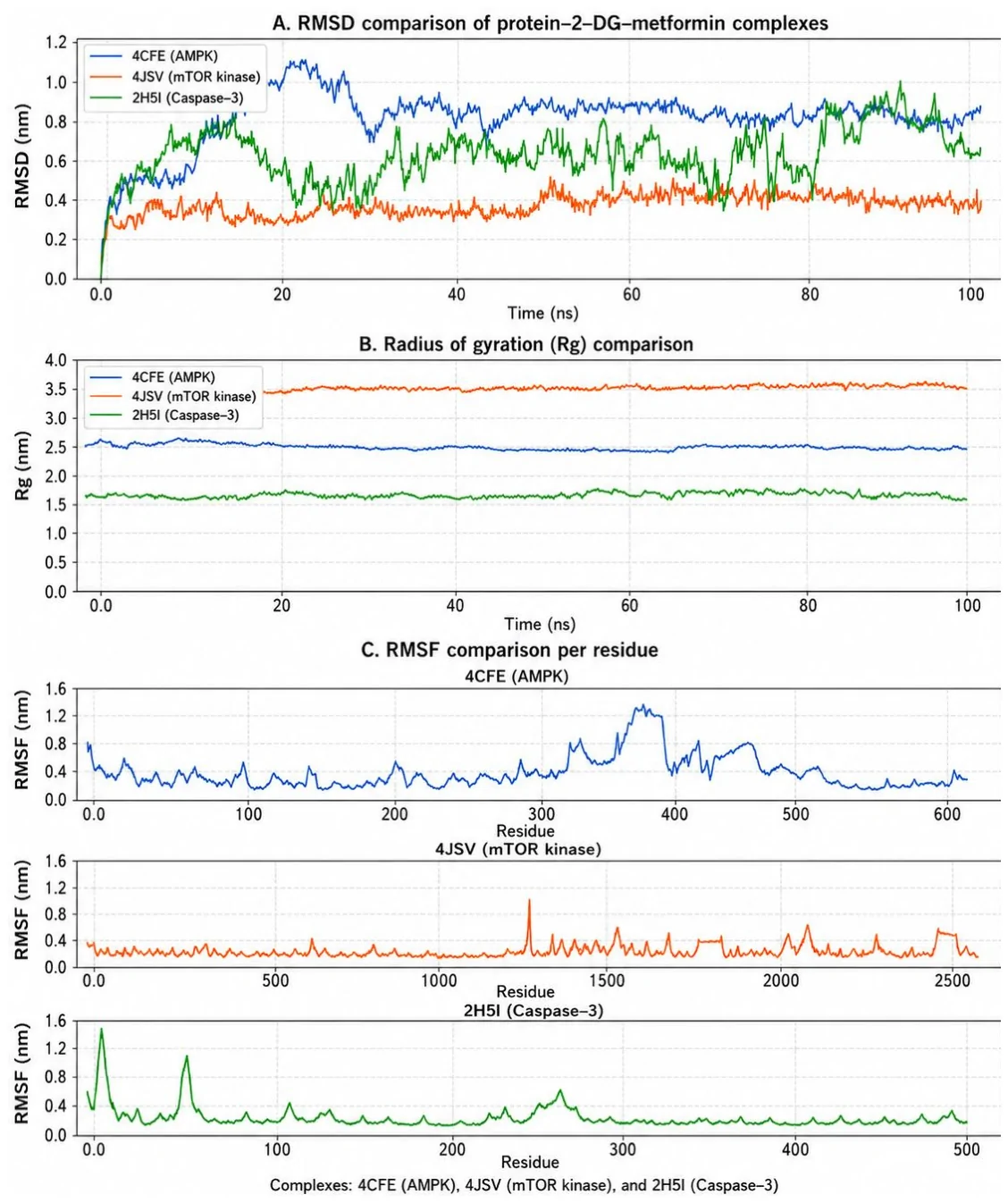
Comparative molecular dynamics analysis of docking-derived protein–2-DG–metformin complexes during 100 ns simulations. (**A**) RMSD profiles of 4CFE (AMPK), 4JSV (mTOR kinase), and 2H5I (caspase-3) complexes during 100 ns MD simulations. (**B**) Radius of gyration (Rg) profiles showing the global compactness of the complexes. (**C**) RMSF profiles of individual residues, shown separately for each complex. The complexes correspond to sequential docking models in which metformin was docked into the corresponding 2-DG-bound protein structure.

To further evaluate the dynamic behavior of the docking-derived ternary complexes, 100 ns MD simulations were analyzed using RMSD, Rg, and RMSF profiles (Figure 4). The RMSD trajectories indicated different levels of global flexibility among the complexes. The 4JSV mTOR kinase complex displayed the lowest average RMSD (approximately 0.36 nm), indicating the highest global stability. These findings suggest that the docking-derived mTOR kinase complex maintained the highest conformational stability during the simulation period. The 2H5I caspase-3 complex showed moderate RMSD fluctuations (approximately 0.62 nm), whereas the 4CFE AMPK complex exhibited higher RMSD values (approximately 0.83 nm), suggesting greater conformational flexibility during the simulation period (Figure 4).

The Rg profiles indicated that the overall compactness of the complexes remained relatively stable during the simulations. The average Rg values were approximately 2.55 nm for 4CFE, 3.62 nm for 4JSV, and 1.76 nm for 2H5I. These differences primarily reflect differences in protein size and structural organization rather than ligand-induced destabilization. No progressive expansion or collapse was observed for any complex, indicating preservation of global compactness (Figure 4).

The RMSF profiles showed that most residues exhibited relatively low fluctuations, whereas higher mobility was restricted to localized flexible regions. The 4CFE AMPK complex displayed increased fluctuations in selected residue regions, consistent with its higher RMSD profile. The 4JSV mTOR kinase complex showed generally low residue-level flexibility with localized peaks, supporting its overall stability. In the 2H5I caspase-3 complex, increased RMSF values were mainly observed in terminal or flexible loop regions, while the remaining residues showed comparatively lower fluctuations (Figure 4).

### 3.4. Combination Index and Dose Reduction Analysis

To investigate whether glycolytic inhibition enhances the antiproliferative activity of metformin, cells were treated with increasing concentrations of metformin in the presence of a fixed concentration of 2-deoxy-D-glucose (2-DG, 1 mM). Combination index (CI) analysis was performed using the Chou–Talalay method based on fraction affected (Fa) values obtained from metformin and 2-DG co-treatment experiments. The calculated CI values are summarized in Table 3, whereas the corresponding Fa values used for CI determination are provided in Supplementary Table S2. Combined treatment resulted in enhanced growth inhibition compared with metformin alone in the examined cancer cell lines. The interaction between metformin and 2-DG was subsequently characterized as synergistic, additive, or antagonistic based on the calculated CI values.

CI analysis indicated that 2-DG enhanced the antiproliferative activity of metformin under selected experimental conditions, although the magnitude of interaction varied depending on cell type and exposure duration. After 48 h, the interaction profile became more variable, with slight antagonistic effects observed in *HeLa* cells and antagonism observed in *A549* cells, whereas MRC-5 cells retained a moderate synergistic response. The CI value for *HT-29* cells could not be reliably determined due to the limited dose range available for analysis.

To quantify the sensitizing effect of glycolytic inhibition on metformin activity, the Dose Reduction Index (DRI) was calculated (Table 4). The strongest apparent sensitization was observed in *HT-29* cells, where DRI values exceeded 26 after 24 h and 10 after 48 h, although these values represent minimum estimates due to the limited concentration range. Although *MRC-5* fibroblasts also showed reduced metformin IC_50_ values after 2-DG exposure, the magnitude of sensitization was lower compared with tumor cell lines, suggesting a greater sensitization of malignant cells under the experimental conditions used.

DRI values indicate the extent to which the metformin dose could theoretically be reduced in the presence of 2-DG to achieve a comparable antiproliferative effect.

## 4. Discussion

Metabolic reprogramming is now recognized as a hallmark of cancer and represents an attractive therapeutic target [1,2,3]. The increased dependence of tumor cells on glucose metabolism, commonly referred to as the Warburg phenotype, creates metabolic vulnerabilities that may be exploited therapeutically [2,3,4]. In the present study, we evaluated the antiproliferative effects of the glycolytic inhibitor 2-deoxy-D-glucose (2-DG) and the mitochondrial complex I inhibitor metformin, administered either alone or in combination, in cervical, lung, and colorectal cancer cell lines. Our findings demonstrate that simultaneous targeting of glycolysis and mitochondrial respiration produces a greater inhibitory effect on cancer cell growth than either agent alone, supporting the concept of dual metabolic targeting as a promising anticancer strategy.

As a single agent, 2-DG induced concentration- and time-dependent growth inhibition in all investigated tumor cell lines, although the magnitude of the response varied considerably. *HeLa* cells were the most sensitive to glycolytic inhibition, displaying the lowest IC_50_ values and the greatest reduction in cell viability following treatment. These findings are consistent with the notion that cervical carcinoma cells exhibit a pronounced dependence on glycolytic ATP production and may therefore be particularly vulnerable to disruption of glucose utilization, although definitive assessment of drug interaction requires full Chou–Talalay dose–response matrices [39,40]. In contrast, *A549* and *HT-29* cells demonstrated a more moderate response, suggesting greater metabolic flexibility and a higher capacity to compensate for glycolytic inhibition through alternative bioenergetic pathways. Such heterogeneity among tumor types is increasingly recognized as a critical determinant of responsiveness to metabolism-based therapies [8,41,42].

The biological effects of 2-DG extend beyond simple ATP depletion. Following cellular uptake, 2-DG is phosphorylated by hexokinase but cannot undergo further metabolism, resulting in inhibition of glycolytic flux and accumulation of metabolic intermediates [19,20,22]. In addition, 2-DG interferes with N-linked glycosylation, promotes endoplasmic reticulum stress, activates the unfolded protein response, and may trigger autophagy. Depending on cellular context, autophagy may function either as a cytoprotective adaptation or as a mechanism contributing to cell death. This duality may partly explain why 2-DG often exhibits only moderate efficacy as a single agent while demonstrating substantially greater activity when combined with agents that simultaneously impair mitochondrial energy production [19,20,22,43].

Metformin also reduced cell viability in a concentration- and time-dependent manner, with *HeLa* cells again displaying the greatest sensitivity. Although metformin is widely recognized as an antidiabetic drug, increasing evidence indicates that it possesses anticancer activity through inhibition of mitochondrial complex I, suppression of oxidative phosphorylation, activation of AMP-activated protein kinase (AMPK), and downstream inhibition of mTOR signaling [14,15,44,45,46]. The clinical relevance of the metformin concentrations used in the present study should be considered. The concentrations applied (0.05–1 mM) are higher than the circulating plasma concentrations typically achieved during standard metformin therapy; however, they are within the range frequently employed in in vitro studies investigating the cellular and molecular mechanisms of metformin action. This discrepancy reflects the need to achieve sufficient intracellular exposure in cell culture models, considering that metformin accumulates in specific tissues, particularly the liver, which represents its primary site of pharmacological activity. Therefore, the concentrations selected in this study were intended to evaluate concentration-dependent cellular responses and mechanistic effects rather than to directly reproduce systemic drug exposure in patients. These effects collectively reduce cellular energy availability and limit anabolic processes required for tumor growth. Importantly, the IC_50_ values obtained in the present study were lower than those reported in several previous investigations, which may reflect differences in cell type, experimental conditions, exposure duration, and methodology used for viability assessment [10,16,45]. Because the SRB assay measures total cellular protein content rather than mitochondrial enzymatic activity, it may provide a more reliable estimate of growth inhibition in studies involving metabolic modulators.

The most significant finding of the present study was the marked enhancement of metformin activity following co-treatment with 2-DG. Simultaneous inhibition of glycolysis and mitochondrial respiration produced substantially greater growth inhibition than either intervention alone and reduced the concentration of metformin required to achieve comparable antiproliferative effects [17,24,27]. This observation was supported by Dose Reduction Index (DRI) analysis, which demonstrated increased sensitivity to metformin in all examined cancer cell lines. Particularly notable was the response of HT-29 cells, where DRI values exceeded 10 following prolonged treatment, indicating pronounced sensitization of HT-29 cells to metformin-mediated growth inhibition. These findings are consistent with previous studies demonstrating that metabolic co-targeting can overcome adaptive resistance mechanisms and induce a state of energetic crisis that exceeds the compensatory capacity of malignant cells.

From a mechanistic perspective, the enhanced activity of the combination can be explained by the complementary modes of action of the two compounds. Cancer cells frequently compensate for inhibition of one metabolic pathway by increasing dependence on another. Metformin limits ATP production through oxidative phosphorylation, whereas 2-DG suppresses glycolytic ATP generation. Simultaneous inhibition of both pathways restricts metabolic plasticity and may lead to profound bioenergetic stress, redox imbalance, impaired biosynthesis, and ultimately growth arrest or cell death [8,17,24,25,26]. The greater efficacy observed following combined treatment therefore supports the concept that metabolic adaptability represents a major determinant of therapeutic resistance and that dual pathway inhibition may be required to achieve meaningful antitumor effects.

An additional finding of potential translational relevance was the comparatively lower sensitivity of normal MRC-5 fibroblasts, particularly at lower treatment concentrations. Although the selectivity observed in vitro requires confirmation in more complex experimental models, these results suggest the existence of a therapeutic window and indicate that malignant cells may be preferentially affected by metabolic interventions. Such selectivity is especially important given the central role of glucose metabolism in normal tissues and remains a key consideration for the clinical development of metabolism-directed therapies [8,16].

The experimental findings of the present study represent the primary evidence supporting the enhanced antiproliferative activity of metformin following 2-DG treatment. In particular, the reduction in metformin IC_50_ values and the increased sensitivity observed in DRI analysis provide direct evidence of functional interaction between the two metabolic interventions. The subsequent molecular docking and molecular dynamics analyses were performed as complementary approaches to explore possible molecular mechanisms underlying these observations. However, these computational findings should be interpreted as supportive and hypothesis-generating rather than as direct confirmation of molecular binding, pathway activation, or pharmacological synergy.

Among the investigated targets, the mTOR kinase model showed the most favorable metformin docking score after 2-DG binding. This is biologically relevant because mTOR signaling represents a central regulator of cell growth, protein synthesis, and metabolic adaptation [47]. However, the relatively large centroid distance between metformin and 2-DG in this complex suggests that the two ligands occupy spatially distinct regions rather than forming a direct co-binding site.

The AMPK complex showed the shortest distance between metformin and 2-DG, indicating the most favorable spatial compatibility between the two ligands. This finding is particularly relevant because AMPK acts as a cellular energy sensor and is closely associated with the metabolic effects of metformin [14,46]. Although docking cannot directly demonstrate pathway activation, the AMPK docking model supports the possibility that both compounds may be accommodated within an energy-sensing protein environment [48]. The caspase-3 model displayed weaker docking energies, but its inclusion is relevant as a downstream apoptosis-related target in the context of metabolic stress-induced growth inhibition and cell death.

The MD simulation results further support the structural persistence of the ternary complexes obtained by docking [49]. The 4JSV mTOR kinase complex displayed the lowest RMSD values and the most stable global behavior, suggesting that the corresponding protein-2-DG-metformin model remained structurally stable during the simulation. The 2H5I caspase-3 complex showed moderate RMSD fluctuations but retained a stable Rg profile, indicating preserved compactness. The 4CFE AMPK complex exhibited higher RMSD values, suggesting greater conformational rearrangement, but its Rg profile remained stable, indicating that these changes did not result in global unfolding or loss of compactness.

Importantly, the docking and MD results should be interpreted as supportive mechanistic evidence rather than as direct proof of pharmacological synergy. The sensitizing effect of 2-DG on metformin activity is primarily supported by the experimental reduction in metformin IC50 values and DRI/CI analysis. The computational results complement these findings by showing that both compounds can be accommodated within biologically relevant protein targets involved in energy sensing, growth regulation, and apoptosis, and that the resulting ternary complexes remain structurally persistent during MD simulations. These findings indicate potentiation of metformin activity by 2-DG; however, complete dose–response combination matrices are required for definitive classification of synergy [17,24].

Several limitations of this study should be recognized. First, all experiments were performed exclusively under in vitro conditions, which do not fully reproduce the biological complexity, metabolic heterogeneity, and cellular interactions of the tumor microenvironment. Consequently, the observed antiproliferative effects may not directly translate to in vivo settings. Second, the molecular mechanisms responsible for the enhanced activity of the combined treatment were not investigated experimentally and should be further elucidated by evaluating apoptosis, autophagy, mitochondrial function, oxidative stress, and key intracellular signaling pathways. Third, although the computational docking and molecular dynamics analyses provide mechanistic support for the experimental findings, these approaches are predictive in nature and should be regarded as hypothesis-generating rather than definitive evidence of molecular interactions. Finally, while the combination consistently potentiated the antiproliferative activity of metformin, formal Chou–Talalay analysis based on complete dose–response matrices was not performed [40,41]. Therefore, the present findings support potentiation of metformin activity by 2-DG but do not allow definitive classification of the interaction as synergistic or additive. Most importantly, the experimental findings were obtained solely from in vitro models, which are unable to fully reproduce the metabolic complexity and dynamic interactions of the tumor microenvironment. Although the combination index (CI) analysis provides a useful quantitative approach for evaluating drug interactions, some limitations should be acknowledged. The CI values are dependent on the experimental conditions, including drug concentrations, exposure time, and the selected mathematical model. Therefore, the observed synergistic effects should be interpreted with caution. Additional combination designs, such as alternative drug ratios, dose–response matrices, and validation in independent experimental models, would further strengthen the assessment of drug interactions and provide a more comprehensive understanding of the underlying pharmacological effects. Future studies employing appropriate in vivo tumor models will be necessary to validate the efficacy, safety, and translational potential of this therapeutic strategy.

## 5. Conclusions

In conclusion, the present study demonstrates that simultaneous targeting of glycolysis and mitochondrial respiration enhances the antiproliferative activity of metformin in multiple cancer cell types. The observed reductions in metformin IC_50_ values and the favorable DRI profiles support the concept that metabolic co-targeting may represent a rational and potentially clinically relevant strategy for improving the efficacy of anticancer therapy. Further mechanistic and in vivo studies are warranted to determine the translational potential of this approach.

## Figures and Tables

**Table 1:** IC_50_ values (mM) of metformin alone and in combination with 1 mM 2-deoxy-D-glucose (2-DG) in *HeLa*, *A549*, *HT-29*, and *MRC-5* cells following 24 h and 48 h treatment.

Cell Line	Met IC_50_ 24 h	Met + 2DG IC_50_ 24 h	Fold Reduction	Met IC_50_ 48 h	Met + 2DG IC_50_ 48 h	Fold Reduction
HeLa	6.04	2.00	3.02	2.28	1.56	1.46
A549	14.79	1.96	7.55	3.30	5.44	0.61
HT-29	26.53	4.26	6.23	10.54	>1	ND ^1^
MRC-5	33.46	7.71	4.34	33.41	18.58	1.80

^1^ IC_50_ values were calculated by nonlinear regression analysis of dose–response curves. Fold reduction represents the decrease in metformin IC_50_ values induced by 2-DG. Data are presented as IC_50_ values (mM). ND, not determined; IC_50_ > 1, value higher than the maximum tested concentration.

**Table 2:** Sequential docking results of metformin in 2-DG-bound protein models associated with cancer metabolism and apoptosis.

PDB ID	Target	Chain	2-DG Binding Energy (kcal/mol)	Met After 2-DG Binding Energy (kcal/mol)
4CFE	AMPK	A	−3.65	−5.02
4JSV	mTOR kinase	A	−3.98	−6.13
2H5I	Caspase-3	A	−3.45	−4.44

**Table 3:** Chou–Talalay combination index (CI) analysis of metformin and 2-deoxy-D-glucose (2-DG) co-treatment in cancer and normal cell lines after 24 h and 48 h exposure.

Cell Line	CI 24 h	Interaction Profile	CI 48 h	Interaction Profile
HeLa	0.51	Enhanced interaction	1.18	Reduced interaction under prolonged exposure
A549	0.33	Marked enhancement of metformin activity	1.83	Reduced interaction
HT-29	0.45	Enhanced interaction	>0.33	Not determined ^1^
MRC-5	0.37	Enhanced interaction	0.77	Moderate interaction

^1^ CI value was not reliably determined due to the limited dose range available for analysis.

**Table 4:** Dose Reduction Index (DRI) of metformin following cotreatment with 2-deoxy-D-glucose (2-DG) in human cancer and normal cell lines.

Cell Line	Time (h)	Metformin IC_50_ (mM)	Metformin + 2-DG IC_50_ (mM)	DRI ^1^
HeLa	24	6.04	2.00	3.02
HeLa	48	2.28	1.56	1.46
A549	24	14.79	<1.96	>7.55
A549	48	3.30	<1.00	>3.30
HT-29	24	26.53	<1.00	>26.53
HT-29	48	10.54	<1.00	>10.54
MRC-5	24	33.46	7.71	4.34
MRC-5	48	33.41	18.58	1.80

^1^ DRI values > 1 indicate that a lower concentration of metformin is required to achieve the same antiproliferative effect in the presence of 2-DG.

## Data Availability

The original contributions presented in this study are included in the article/Supplementary Materials. Further inquiries can be directed to the corresponding author.

## Supplementary Materials

The following supporting information can be downloaded at: https://www.mdpi.com/article/10.3390/cancers18162537/s1, Table S1: IC_50_ values (mM) of 2-deoxy-D-glucose (2-DG) and metformin in *HeLa*, *A549*, *HT-29*, and *MRC-5* cells following 24 h and 48 h treatment. IC_50_ values were calculated by nonlinear regression analysis of dose–response curves. Table S2: Fraction affected (Fa) values obtained from metformin and 2-deoxy-D-glucose (2-DG) combination treatment in human cancer and normal cell lines, used for Chou–Talalay combination index calculations.
