# Supplementary material for: Dual Metabolic Targeting of Cancer: Lessons from Metformin and 2-Deoxy-D-Glucose Combinations
Source: Cancers (Basel). 2026 Aug 7;18(16):2537. doi: 10.3390/cancers18162537 (PMC13510935; doi:10.3390/cancers18162537)
Supplement: Supplementary file 1 [file cancers-18-02537-s001.zip › cancers-4461075-supplementary.pdf]

Supplementary Materials for

Dual Metabolic Targeting of Cancer: Lessons from Metformin and 2-Deoxy-D-Glucose Combinations

Vesna Zeljković [1,*], Slaviša Minić [1], Marko Mladenović [2,3], Dejan Milenković [4], Zoran Marković [2], Tanja V. Soldatović [2], Vanja Kunkin [5] and Maja Karaman [6]

[1] Department of Biomedical Sciences, State University of Novi Pazar, Vuka Karadžića 9, 36300 Novi Pazar, Serbia; sminic@np.ac.rs
[2] Department of Sciences and Mathematics, State University of Novi Pazar, Vuka Karadžića 9, 36300 Novi Pazar, Serbia; markohem87@gmail.com (M.M.); zmarkovic@uni.kg.ac.rs (Z.M.); tsoldatovic@np.ac.rs (T.V.S.)
[3] Department of Chemistry, Faculty of Sciences and Mathematics, University of Niš, Višegradska 33, 18000 Niš, Serbia
[4] Department of Science, Institute of Information Technologies, University of Kragujevac, Liceja Kneževine Srbije 1A, 34000 Kragujevac, Serbia; dejanm@uni.kg.ac.rs
[5] Đorđe Joanović Zrenjanin General Hospital, Dr Vase Savića br. 5, 23000 Zrenjanin, Serbia; kunkinbre@gmail.com
[6] Department of Biology and Ecology, Faculty of Sciences, University of Novi Sad, Trg Dositeja Obradovića 2, 21000 Novi Sad, Serbia; maja.karaman@dbe.uns.ac.rs
* Correspondence: vzeljkovic@np.ac.rs

Table S1. IC_{50} values (mM) of 2-deoxy-D-glucose (2-DG) and metformin in *HeLa*, *A549*, *HT-29*, and *MRC-5* cells following 24 h and 48 h treatment. IC_{50} values were calculated by nonlinear regression analysis of dose–response curves.

Cell line.	24h IC_{50}	48h IC_{50}
**HeLa**	**2.0**	**1.56**
**A549**	**1.96**	**5.44**
**HT29**	**4.26**	**>1**
**MRC5**	**7.71**	**18.58**

Table S2. Fraction affected (Fa) values obtained from metformin and 2-deoxy-D-glucose (2-DG) combination treatment in human cancer and normal cell lines, used for Chou–Talalay combination index calculations.

Cell line	Fa (24 h, 1 mM Met)	Fa (48 h, 1 mM Met)
HeLa	0.366	0.446
A549	0.093	0.383
HT29	0.088	0.353
MRC-5	0.123	0.362
